# Supplementary material for: Has the COVID-19 Pandemic Changed Parental Attitudes and Beliefs Regarding Vaccinating Their Children against the Flu?
Source: Vaccines (Basel). 2023 Sep 24;11(10):1519. doi: 10.3390/vaccines11101519 (PMC10611412; doi:10.3390/vaccines11101519)
Supplement: Supplementary file 1 [file vaccines-11-01519-s001.zip › vaccines-2583212-supplementary.pdf]

Supplementary Tables

Supplementary Table S1. Items and internal consistency for assessing measures of the HBM

Measures	Items	a
Perceived susceptibility	I believe that if I my children will get vaccinated, the likelihood of them getting infected with Flu will decrease	0.88
	I believe that if my children will not get vaccinated, the likelihood of our family and relatives getting infected with Flu will increase	
*Perceived severity	Even if my children will get infected with Flu I do not think it will cause them significant suffering or complications	0.64
	Even if my children will get infected with Flu, the likelihood of them recovering from the disease is very high	
Perceived Benefits	I believe that the Flu vaccine will be highly effective in preventing my children from experiencing significant complications of the disease	0.92
	I believe that if my children will get vaccinated against Flu, the likelihood that they will miss school days (school / kindergarten) will decrease	
	I believe that if my children will get vaccinated against Flu, the likelihood of us (the parents) losing work days will decrease	
Perceived barriers	I am afraid that the Flu vaccine has side effects	-
Cues to action	The likelihood of vaccinating my children against Flu will increase if the health minister will express their support for the benefits of vaccinating children	0.88
	The likelihood of vaccinating my children against Flu will increase if my pediatrician will recommend that they be vaccinated	
	The likelihood of vaccinating my children against Flu will increase if the vaccine will be administered by the educational system	
Attitude	I support vaccine and My children are vaccinated according to the standard immunization schedule	-

a Cronbach indicates the internal consistency: **HBM** a= 0.86
Items response scale: 1-6 agreement
* Negative items were reverse scored

Supplementary Table S2: Comparing the sample and the entire population in terms of the characteristics used for sampling: age, gender, level of religiosity, and geographical area

**Sociodemographic**	**Population**	**sample (n=1012)**	
	%	N	%
**Age group ***			
18-39	58%	617	63%
40-60	42%	358	37%
**Gender ***			
Male	50%	467	48%
Female	50%	505	52%
**Level of religiosity ****			
Secular	45%	417	43%
Traditional	25%	247	25%
Religious	16%	128	13%
Haredi	14%	101	10%
**Geographical area ****			
Jerusalem District	11%	93	9%
Northern District	9%	124	12%
Haifa District	11%	159	16%
Central District	29%	271	27%
Tel Aviv District	20%	168	17%
Southern District	14%	146	14%
Judea and Samaria Area	6%	48	5%

* Jewish population, ages 18-60 (n=3.4 million)
** Jewish population, all ages (n=6.4 million)

Supplementary Table S3: Reasons for the reluctancy to vaccinate children against the flu (n = 967 reasons provided by the 435 parents didn’t vaccinate and don’t intend to vaccinate their children against the flu in the winter of 2023

Reasons for reluctancy to vaccinate children against the flu	n	%
**I am afraid of the side effects of the vaccine**	272	28%
I believe that the vaccine is not effective	212	22%
The natural vaccine protects more than an injected vaccine	164	17%
Lack of trust in the Ministry of Health	128	13%
Lack of trust in pharmaceutical companies and vaccines	128	13%
I am against all types of vaccines in general	29	3%
Inaccessibility	21	2%
Medical contraindication (sensitivity to the vaccine)	13	1%

Respondents could provide more than one reason for the reluctance to vaccinate children against the flu in the winter of 2023. Hence, the number of reasons is larger than the number of respondents
